# The Possibility of Digital Imaging in the Diagnosis of Occlusal Caries

**DOI:** 10.1155/2010/860515

**Published:** 2010-03-07

**Authors:** Sachi Umemori, Ken-ichi Tonami, Hiroshi Nitta, Shiro Mataki, Kouji Araki

**Affiliations:** ^1^General Dentistry, Graduate School, Tokyo Medical and Dental University, 1-5-45 Yushima, Bunkyo-ku, Tokyo 113-8549, Japan; ^2^Oral Diagnosis and General Dentistry, Dental Hospital, Tokyo Medical and Dental University, 1-5-45 Yushima, Bunkyo-ku, Tokyo 113-8549, Japan; ^3^Behavioral Dentistry, Department of Comprehensive Oral Health Care, Graduate School, Tokyo Medical and Dental University, 1-5-45 Yushima, Bunkyo-ku, Tokyo 113-8549, Japan; ^4^Center for Education Research in Medicine and Dentistry, Tokyo Medical and Dental University, 1-5-45 Yushima, Bunkyo-ku, Tokyo 113-8549, Japan

## Abstract

The aim of this study was to assess the possibility of digital image analysis of pit-and-fissure discoloration in order to diagnose caries. Digital images showing pit-and-fissure discoloration in 100 teeth of 19 patients were analyzed to obtain the fractal dimension (FD) and the proportion of the area of pit-and-fissure discoloration to the area of occlusal surface (PA). DIAGNOdent values were measured (DD), and dentists' diagnoses were also obtained. The sensitivity and specificity of FD, PA, DD, and the combination of FD and PA compared to the dentists' diagnoses were calculated. The sensitivities of FD, PA, DD, and the combination of FD and PA were 0.89, 0.47, 0.69, and 0.86, respectively, and the specificities were 0.84, 0.95, 0.91, and 0.86, respectively. Although further research is needed for the practical use, it is possible to use the analysis of digital images of pit-and-fissure molar discoloration as a diagnostic tool.

## 1. Introduction

In recent years, the concept of minimal intervention (MI) has prevailed in dentistry. MI can be defined as the maximum preservation of the healthy dental structure [1]. Therefore, the importance of diagnosing caries at an early stage has increased. In conventional procedures, the diagnosis of caries has mainly consisted of visual inspection and tactile assessment with probing. However, Lussi [2] reported that the sensitivity of detecting caries was 0.62 by visual inspection and 0.82 by probing. In addition, the pressure of probing can damage the demineralized fissure and increase the risk that caries progress [3, 4]. To promote MI, diagnosis without a probe has been recommended [3]. The laser fluorescence-based caries detection device DIAGNOdent (Kavo, Germany) has been introduced as an alternative. However, no single detection method for caries is sufficient; therefore, the combination of some detection methods has been recommended [5–7].

Recent improvements in the personal computer have made the process of digital imaging more efficient and convenient [8]. Nevertheless, few applications use the quantitative evaluation of digital images to diagnose caries. If the shape of caries can be quantified, and the relationship between the numerical value and the condition of the lesion can be demonstrated, this information would be helpful to diagnose dental caries. One of the indexes which evaluates shape quantitatively is fractal dimension. The authors of this paper have previously shown that the fractal dimension and proportion of the area of pit-and-fissure discoloration to the area of occlusal surface obtained by digital imaging were significantly correlated with the depth of the caries and the DIAGNOdent values in extracted teeth [9]. For assessment of the method as a diagnostic system, the ability of the diagnosis, such as the sensitivity, the specificity, and the accuracy, should be researched in clinical situation. The aim of this study was to assess the possibility of the clinical application of the diagnosis of occlusal caries using digital imaging by examining the sensitivity, the specificity, and the accuracy in comparison with the DIAGNOdent values and the dentists' diagnoses.

## 2. Materials and Methods

One hundred teeth (36 premolars and 64 molars) with pit-and-fissure discoloration from 19 outpatients were examined at the Clinic of Oral Diagnosis and General Dentistry, Dental Hospital, Tokyo Medical and Dental University. The occlusal surface of each tooth was washed with the Robinson brush to remove dental plaque without any abrasive paste. Then, pit-and-fissure discoloration was dried by air and measured three times using DIAGNOdent. The mean scores were used as the DIAGNOdent values of the teeth (DD). Next, the occlusal surface of each tooth was photographed as large as possible with an intraoral digital camera (Penscope, Morita, Japan). Each image was stored in a personal computer using a video capture interface (PC-MDVD/U2, Buffalo, Japan). Without knowing the DD, a dentist preliminarily diagnosed each tooth using visual inspection and tactile examination to decide which treatment plan would be appropriate (preventive or operative). The clinical diagnosis of preventive treatment for teeth was classified as CO. On the other hand, carious lesions requiring operative treatment were removed in a conventional clinical way. If the resulting cavity preparation was limited in the enamel, then the clinical diagnosis was classified as C1. If the resulting cavity preparation reached the dentin, and sound tissue still remained between the cavity and the pulp chamber, then the clinical diagnosis was classified as C2. No lesion reached the pulp chamber in this study. Five dentists ranging from 3 to 15 years of professional experience examined the teeth after calibration of the criteria of the caries assessment conducted before the study; the calibration was done as follows; first, the five dentists examined 30 extracted teeth and decide which treatment plan would be appropriate (preventive or operative). At that time, the rate of accordance among the five dentists was 76.7%. Then, the teeth were sliced pallarel to the teeth axis and to the depth of lesion for each tooth was determined. At last, the dentists discussed to accord the treatment planning for each tooth referring the depth of the lesion. 

The digital photographs obtained were processed and analyzed using image analysis software (Image J, NIH, USA). First, each image was converted to an 8-bit gray-scale image, in which the density of grayness of each pixel was linearly scaled from min 0 (black) to max 255 (white). Then, the occlusal surface in the image was isolated from the background using a density histogram of the image, and the area was measured. Pit-and-fissure discoloration was also isolated from the occlusal surface using the density histogram, and the area was measured. The proportion of the area of pit-and-fissure discoloration to the area of the occlusal surface was calculated (PA). Next, the image of the isolated pit-and-fissure discoloration was converted into a binary image, in which the density of pit-and-fissure discoloration was 0, and its background was 255, followed by calculating the fractal dimension of pit-and-fissure discoloration (FD). 

Differences in FD, PA, and DD between each clinical diagnosis were analyzed using two-way ANOVA and Games-Howell test to reveal the clinical diagnosis and the effect of the examining dentists. The correlation between the clinical diagnosis and each FD, PA, and DD was analyzed using Spearman's correlation coefficient. Discriminant formulas were obtained using discriminant analysis with the treatment plan (preventive/operative) as the objective variable and FD, PA, and DD as explanatory variables. Sensitivity and specificity were calculated by applying FD, PA, and DD to each discriminant formula. The accuracy, ratio of the number of teeth showing accordance between the treatment plan decided by the dentists and the predictive treatment plan decided using the discriminant formula to the number of all the teeth, was also obtained. All the statistical analyses were performed using SPSS 16.0 (SPSS Inc., USA). The entire process was approved by the Ethics Committee of the Faculty of Dentistry, Tokyo Medical and Dental University (No. 317).

## 3. Results

FD, PA, and DD values corresponding to each clinical diagnosis are shown in Table 1. FD, PA, and DD increased with the depth of the caries. The two-way ANOVA revealed that the FD, PA, and DD were different among the clinical diagnosis (*P* < .01). On the other hand, the difference of the examining dentists did not affect the FD, PA, and DD. Spearman's correlation coefficients between the clinical diagnosis and each FD, PA, and DD were 0.743, 0.700, and 0.652, respectively (*P* < .01). There were also significant correlations among FD, PA, and DD (*P* < .01).

Table 2 shows the discriminant formula, sensitivity, specificity, and accuracy of each explanatory variable. Based on the discriminant formula of each explanatory variable, the thresholds of FD, PA, and DD between preventive and operative treatments were 1.20, 0.012, and 28.8, respectively. The sensitivity of FD was greater than that of PA, DD, and the combination of FD and PA. The specificity of PA was greater than that of FD, DD, and the combination of FD and PA. The accuracy of the combination of FD and PA was greater than that of FD, PA, and DD.

## 4. Discussion

Previously, it was reported that the fractal dimension and the proportion of the area of pit-and-fissure discoloration to the area of occlusal surface were significantly correlated with the depth of the caries and the DIAGNOdent values in extracted teeth [9]. In this study, the same tendency was observed for patients' intraoral teeth. The fractal dimensions for C0, C1 and C2 in the former study were 0.97, 1.30, and 1.52, respectively [9]. These results indicate that image analysis of molar pit-and-fissure discoloration was clinically useful for the diagnosis of caries. An increase of the proportion of the area of discoloration corresponded to a change of the volume of caries lesion, while an increase of the fractal dimension corresponded to a change of the shape of the lesion caused by caries progression. 

A fractal is a geometric shape, possessing characteristics of self-similarity or self-affinity, and widely observed in nature [10, 11]. Recently, fractals have been in the spotlight in the field of medicine, and research has been introduced regarding its use in the field of diagnosis [12–14]. Fractal dimension, is a quantifiable value that characterizes shape. The dimension increases in number with the complexity of the structure. For example, a point is described as the zero dimension; a straight line is described as the first dimension, and a plane is described as the second dimension. The fractal dimension is a decimal dimension between integers. Such decimal dimensions can be obtained by expanding the definition of the dimension as the rate at which the perimeter (or the surface area) of an object increases, and the measurement scale is reduced [10]. Several ways to measure the fractal dimension have been introduced. In this study, the authors used a simple way to determine the fractal dimension called box counting. In this method, a grid of squares is placed over the object, and the number of squares through which any part of the object passes is counted. This process is repeated with different grids having different sizes. The number of squares placed over the object versus the length of the side of the square are then plotted on log-log scale. When a regression line is obtained from the plots, the slope of the line is defined as the dimension. The degree of uneven complexity of a boundary or a coast can be quantified using this approach. In this research, the fractal dimension of discoloration increased from 0.8 to 1.6 as the depth of the caries increased, which corresponded to a change in the shape of the discolored area from a point or a line to an area based on the progression of the caries. 

The sensitivity and the accuracy of FD were greater than that of DD. The sensitivity of PA was less than that of FD and DD. Generally, the addition of valuables into discriminant formula is one of the ways to improve the accuracy, however, in this study, the accuracy of the combination of FD and PA was similar to that of single FD. Therefore, further study to find other valuables is needed to improve the accuracy of this method by combination of valuables. Thus, the accuracy of the diagnosis of occlusal caries using digital images of discolored areas was comparable to that of DIAGNOdent; therefore, its clinical application as a diagnostic tool is possible. 

Because this study was clinical, the final diagnosis of each examined tooth was not confirmed by a histological procedure but by a dentist's clinical examination. As mentioned above, the diagnoses of dentists were reported to vary [2]. In this study, the results of two-way ANOVA showed that the effect of the examining dentist on the DD, PA, and FD values was not significant. Therefore, we considered that difference of the diagnosis among the dentists would be small. 

In the present study, to examine the possibility of digital imaging in the diagnosis of occlusal caries, DIAGNOdent was used as a comparative pre-existing dental caries detection tool. There are other caries detection tools, such as fiber optic transillumination (FOTI), digital imaging fiber optic transillumination (DIFOTI), quantitative laser or light fluorescence (QLF), and electrical conductive measurements (ECM). FOTI, DIFOTI, and QLF have been tested in vivo, however, the number of clinical studies has still been small [15, 16]. On the other hand, a comparatively long time has passed since DIAGNOdent was introduced to the market, and many findings have been reported. Sheehy et al. [17] reported that DIAGNOdent had greater sensitivity and specificity than ECM. The correlation coefficient between DIAGNOdent readings and the depth and the volume of caries lesions was reported to be 0.47 [18]. Several researches have pointed out that DIAGNOdent measurements are affected by other factors, such as hypomineralization, plaque, debris, staining and wetness [19–21], while a high correlation between inter and intraobserver agreements was also mentioned [22, 23]. We employed DIAGNOdent as a comparison because the diagnosis was provided as a number, the handling was easy, and, moreover, its clinical use has been discussed in other studies. 

In the present study, diagnosis using digital images of pit-and-fissure discoloration depended on the statistical relationship between the shape of discoloration and the depth of caries. Namely, the method did not measure infected tissue of individual teeth directly. The shape of the discolored area in the occlusal surface is, however, possibly affected by medication history, individual history, and lifestyle [6]. Consequently, diagnosis using digital images of pit-and-fissure discoloration is rather experimental. Additionally, the procedure cannot be applied to colorless lesions, such as acute caries. Therefore, diagnosis using digital images of pit-and-fissure discoloration should not be used for definitive diagnosis. Rather, initial diagnosis, screening such as mass examination would be suitable because of its good sensitivity of FD and, the convenience of the procedure. Actually, the core of the diagnostic system is digital imaging processing by computers. If computer programing for all procedures was achieved, then screening of hundreds of examinees would be automated after photograph taking, which would make mass examination less time-consuming with low cost. For such automated uses of the computer, the process of extracting colors from the image must be improved; in this study, the threshold for the colored area was decided one by one by an observer's visual inspection. How to calculate the fractal dimension is also open to discussion. We used the box-counting method attached in the image analysis software, IMAGE J. The box-counting method is only suitable for self-similar profiles, not for more general, self-affine cases [10], that is, the fractal dimension using the box-counting method might be the approximate value. A special computer program must be developed to measure the fractal dimension more accurately, for example, the Minkowski method or the Richardson method [8, 10]. As mentioned above, the shape of the discolored area on an occlusal surface is affected by many factors. Therefore, the thresholds or the discriminant formulas acquired from this research are not universal. Further research is needed to determine the discriminant formulas to diagnose caries using the image analysis of molar pit-and-fissure discoloration.

## Figures and Tables

**Table 1:** FD, PA, and DD of each clinical diagnosis.

Clinical	*n*	FD	PA	DD
diagnosis		(SD)	(SD)	(SD)
C0	64	1.09^a^	0.005^b^	16.9^cd^
		(0.16)	(0.009)	(15.0)
C1	24	1.34^a^	0.012^b^	45.2^c^
		(0.09)	(0.008)	(25.1)
C2	12	1.52^a^	0.051^b^	57.9^d^
		(0.09)	(0.022)	(27.7)

a, b, c, d: Numbers with the same superscript letters are significantly different (*P* < .01).

**Table 2:** The results of discriminant analysis.

Explanatory variables	Discriminant formula	Sensitivity	Specificity	Accuracy
FD	*Y* = 6.74 FD − 8.12	0.89	0.84	0.86
PA	*Y* = 63.3 PA − 0.77	0.47	0.95	0.78
DD	*Y* = 0.05 DD − 1.44	0.69	0.91	0.83
FD, PA	*Y* = 5.68 FD + 17.8 PA − 7.29	0.86	0.88	0.87
